# 

**Published:** 1895-02-16

**Authors:** 


### CONTENTS.

**Boarding Schools as Disseminators of Disease** - - 341

**Micro-Organisms of Sewage** - - - - - - - - 342

**The Noises of Great Cities** - - - - - - - - 342

**On Modern Progress in Ophthalmic Medicine and Surgery.** By ROBERT BRUDENELL CARTER, F.R.C.S.—Diseases of the Cornea - - - - - - - - - 343

**Notes and News** - - - - - - - - - - 346

**Medical Progress and Hospital Clinics:**

Localised Œdema - - - - - - - - - - 347

PROGRESS IN SURGERY.—Orthopædic Surgery - - - - 348

PROGRESS IN GYNÆCOLOGY - - - - - - - - 350

THERAPEUTIC NOTES - - - - - - - - - 352

NEW APPLIANCES AND THINGS MEDICAL - - - - - 352

**Darwinism and Race Progress** - - - - - - - 344

**Annotations.**—Infectious Catarrh—Mr. Balfour's Belief—Sewage Farming in India - - - - - - - - - - 345

**The Institutional Workshop:**

HOSPITAL CONSTRUCTION.—The Livingstone Cottage Hospital, Dartford - - - - - - - - - - - 353

HOSPITAL ADMINISTRATION.—Derbyshire Royal Infirmary—Cottage Hospitals and General Practitioners—Miss Shannon and the Grantham Infirmary - - - - - - - 354

PRACTICAL DEPARTMENTS.—Children's Cots - - - - - 355

HOSPITAL FESTIVALS AND MEETINGS - - - - - - 355

**Scraps and Gleanings** - - - - - - - - - 356

**The Book World of Medicine and Science** - - - - 357

### EXTRA SUPPLEMENT.—THE NURSING MIRROR.

**News from the Nursing World.**—Contemplated Improvements—The Lewisham Guardians—A Parsee Hospital—The Mildmay Mission Hospital—A Training Hospital—What does a Badge Guarantee?—Difficulties in District Nursing—Pauperised or Protected?—Provision for Barry—Vaccination Vigorously Advocated — Hard Lives — Short Items - - - - - - - - - - cxlviii

**Probationers and Junior Nurses** - - - - - - cl

**Elementary Anatomy and Surgery for Nurses.** By W. MCADAM ECCLES, M.B., M.S., F.R.C.S. VI.—Prevention of Suppuration—Antiseptic and Aseptic Surgery - - cxlix

**Where to Go** - - - - - - - - - - cl

**Notes and Queries—Wants and Workers** - - - - cl

**Dress and Uniforms.**—Charing Cross Hospital - - - cli

**The Book World for Women and Nurses** - - - - clii

**For Reading to the Sick** - - - - - - - clii

